# *catena*-Poly[[chlorido[1-(3-nitro­phen­yl)-2-(triphenyl­phospho­ranyl­idene)ethanone-κ*C* ^2^]mercury(II)]-μ-chlorido]

**DOI:** 10.1107/S1600536812043073

**Published:** 2012-10-24

**Authors:** Alireza Dadrass, Jabbar Khalafy, Hojjatollah Rahchamani, Hassan Nasri Koureh, Hooman Yaghoobnejad Asl

**Affiliations:** a Department of Chemistry, Faculty of Science, Urmia University, PO Box 57153-165, Urmia, Iran; b Department of Chemistry, Missouri University of Science and Technology, Rolla, MO 65409, USA

## Abstract

In the title organometallic polymer, [HgCl_2_(C_26_H_20_NO_3_P)]_*n*_, the monodentate 1-(3-nitro­phen­yl)-2-(triphenyl­phospho­ran­yl­­idene)ethanone ligand is coordinated to the Hg^II^ atom through the methine C atom. The Hg^II^ atom is four-coordinated in a distorted tetra­hedral geometry by one terminal Cl atom, two bridging Cl atoms, and one C atom from the ylidic ligand, resulting in a polymeric chain parallel to [010]. The terminal Cl atom is more strongly bound to the Hg^II^ ion [2.3916 (9) Å] than the bridging Cl atoms. The bridge is asymmetric, as indicated by the two different Hg—Cl(bridging) bond lengths [2.5840 (8) and 2.7876 (8) Å]. Intra­molecular C—H⋯O and weak C—H⋯Cl contacts stabilize the polymeric chain. In the crystal, adjacent chains inter­act *via* C—H⋯O hydrogen bonds.

## Related literature

For an example of a one-dimensional polymeric Hg^II^ complex, see: Ebrahim *et al.* (2007 ▶). For mono- and dimeric complexes of Hg^II^ containing ylide ligands, see: Sabounchei *et al.* (2007 ▶, 2008 ▶, 2009 ▶, 2011 ▶); Sabounchei, Jodaian *et al.* (2010 ▶); Sabounchei, Samiee *et al.* (2010 ▶). 
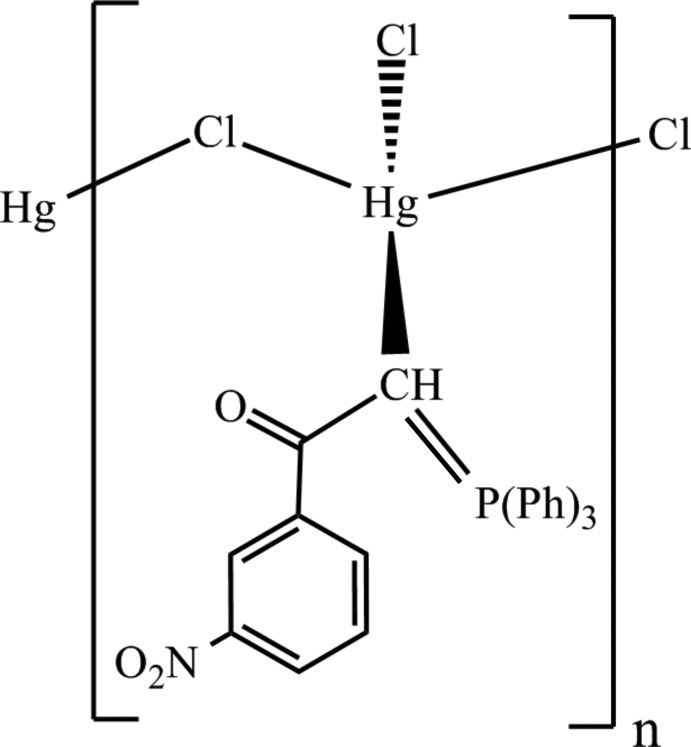


## Experimental

### 

#### Crystal data

[HgCl_2_(C_26_H_20_NO_3_P)]
*M* *_r_* = 696.89
Monoclinic, 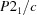

*a* = 12.589 (3) Å
*b* = 8.1026 (17) Å
*c* = 25.231 (6) Å
β = 105.174 (2)°
*V* = 2483.8 (9) Å^3^
*Z* = 4
Mo *K*α radiation
μ = 6.51 mm^−1^
*T* = 293 K
0.61 × 0.15 × 0.12 mm

#### Data collection

Bruker SMART APEX CCD area-detector diffractometer
Absorption correction: multi-scan (*SADABS*; Bruker, 2008 ▶) *T*_min_ = 0.109, *T*_max_ = 0.499
29804 measured reflections
6143 independent reflections
5049 reflections with *I* > 2σ(*I*)
*R*_int_ = 0.034

#### Refinement

*R*[*F* ^2^ > 2σ(*F* ^2^)] = 0.025
*wR*(*F* ^2^) = 0.067
*S* = 1.03
6143 reflections
307 parameters
H-atom parameters constrained
Δρ_max_ = 0.96 e Å^−3^
Δρ_min_ = −0.45 e Å^−3^

### 

Data collection: *SMART* (Bruker, 2002 ▶); cell refinement: *SAINT* (Bruker, 2008 ▶); data reduction: *SAINT*; program(s) used to solve structure: *SHELXS97* (Sheldrick, 2008 ▶); program(s) used to refine structure: *SHELXL97* (Sheldrick, 2008 ▶); molecular graphics: *SHELXTL* (Sheldrick, 2008 ▶); software used to prepare material for publication: *SHELXTL*.

## Supplementary Material

Crystal structure: contains datablock(s) I, global. DOI: 10.1107/S1600536812043073/bh2457sup1.cif

Structure factors: contains datablock(s) I. DOI: 10.1107/S1600536812043073/bh2457Isup2.hkl

Additional supplementary materials:  crystallographic information; 3D view; checkCIF report

## Figures and Tables

**Table 1:** Hydrogen-bond geometry (Å, °)

*D*—H⋯*A*	*D*—H	H⋯*A*	*D*⋯*A*	*D*—H⋯*A*
C10—H10*A*⋯O3	0.93	2.33	3.118 (3)	142
C6—H6*A*⋯Cl1	0.93	2.83	3.630 (3)	145
C24—H24*A*⋯O3^i^	0.93	2.39	3.206 (3)	146

Symmetry code: (i) 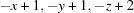.

**Fig. 1.**
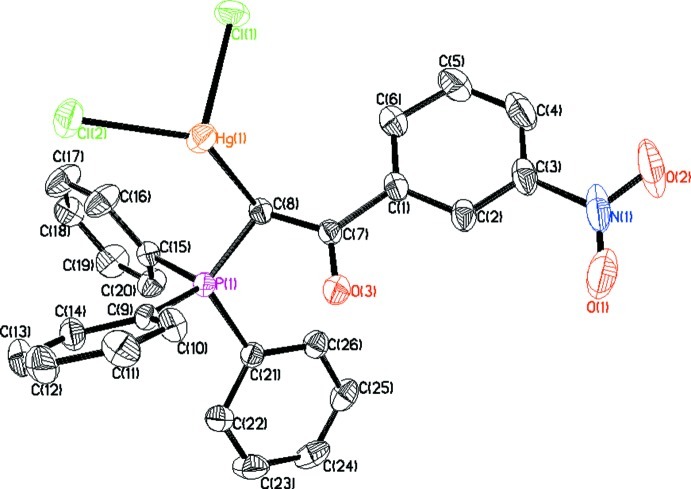
The building unit of the title complex [HgCl2(L)]n. All H atoms have been omitted for clarity. Displacement ellipsoids are drawn at the 50% probability level.

**Fig. 2.**
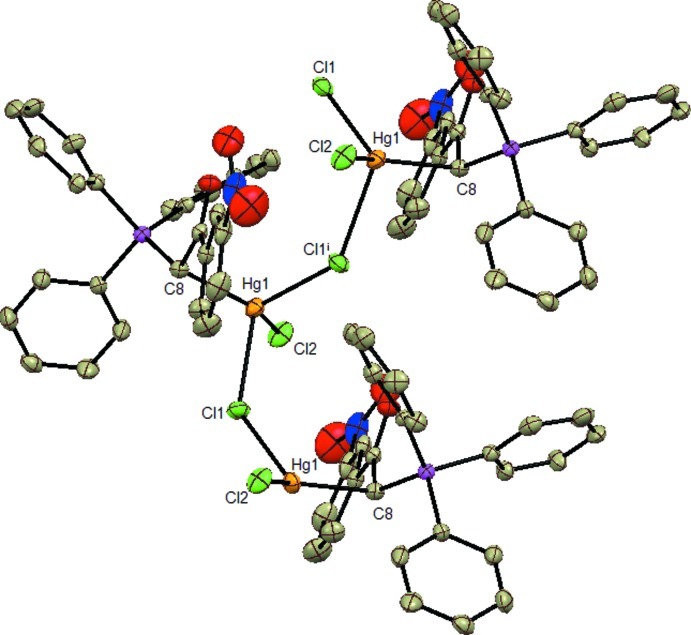
Part of the polymeric chain in the title compound. All H atoms have been omitted for clarity.

**Fig. 3.**
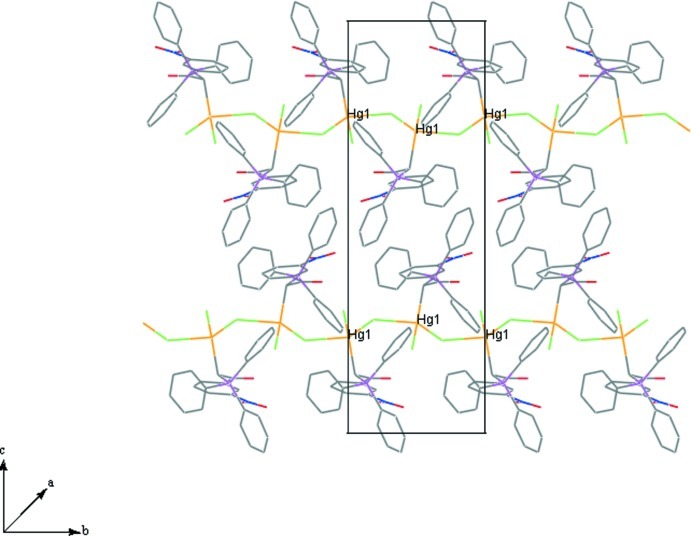
A packing diagram for the title compounds, with chains running parallel to the b axis. All H atoms have been omitted for clarity.

**Fig. 4.**
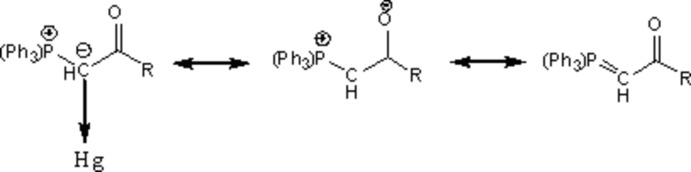
Resonance forms of the phosphorus ylide(ligand) and its coordination with mercury ion.

## supplementary crystallographic information

### Comment

The coordination chemistry of ambidentate ligands (*L*) has been investigated. The preparation and characterization of stabilized phosphorus ylides, and metal complexes incorporating this type of ylides, has attracted much attention in recent years (Ebrahim *et al.*, 2007). Although many bonding modes are possible for keto-ylides, coordination through ylides methine carbon is more predominant and observed with soft metal ions, *e.g*. Pd(II), Pt(II), Hg^II^ and Au(I), whereas O-coordination dominates when the metals involved are hard, *e.g*. Ti(IV), Zr(IV), and Hf(IV). The crystal and molecular structure of the title complex reveals a polymeric structure, with Hg^II^ ions linked by bridging chlorine, participating in a very asymmetric linear bridge. The asymmetric ligand plays an important role in the building of this unusual supramolecular structure. The building unit (Fig. 1) is repeated in the polymeric crystal structure. The polymeric structure (part of the chain) and packing diagram for the complex are shown in Fig. 2 and Fig. 3, respectively. The X-ray analysis reveals the coordination of the ylide ligand through the methine C atom only. The Hg^II^ atom is located in a distorted tetrahedral environment, with one terminal Cl^-^ and two bridging Cl^-^ ions, and one C atom from the ligand, resulting in a one-dimensional polymeric chain. The bond angles around Hg^II^ ion indicate a severe distortion from ideal tetrahedral geometry. The Hg—Cl(terminal) bond length, 2.3916 (9) Å, is shorter than Hg—Cl(bridging) distances. The asymmetric bridging nature of the two chloro ligands is reflected in the unequal Hg—Cl distances of 2.5840 (8) and 2.7876 (8) Å. The latter distance being rather long, indicates a weak Hg—Cl(bridging) interaction. The Hg—C bond length in the title compound, 2.244 (3) Å, is consistent with values reported for mono ([HgI_2_(*bbtppy*)(DMSO)]) and dimeric ([(*bappy*)HgI_2_]_2_) Hg^II^—C complexes (Sabounchei *et al.*, 2007, 2008, 2009, 2011; Sabounchei, Jodaian *et al.*, 2010; Sabounchei, Samiee *et al.*, 2010). In the crystal structure, adjacent polymer chains interact *via* C—H···O hydrogen bonds. Intramolecular C—H···O and weak C—H···Cl hydrogen bonds stabilize the polymeric chains.

### Experimental

All reagents and solvents employed were commercially available and were used as received without further purification. The ligand 1-(3-nitrophenyl)-2-(triphenylphosphoranylidene)ethanone (0.637 g, 1.5 mmol) in methanol (10 ml) was added dropwise to HgCl_2_ (0.407 g, 1.5 mmol) in the same solvent (10 ml). After standing for 15 h without stirring at room temperature, colourless crystals of the title complex were obtained. Yield: 62%.

### Refinement

H atoms were positioned geometrically with C—H = 0.93 Å (aromatic) or 0.98 Å (methine) and constrained to ride on their parent atoms, with *U*_iso_(H) = 1.2*U*_eq_(carrier C).

### Crystal data

**Table:**

[HgCl_2_(C_26_H_20_NO_3_P)]	*F*(000) = 1344
*M**_r_* = 696.89	*D*_x_ = 1.864 Mg m^−^^3^
Monoclinic, *P*2_1_/*c*	Mo *K*α radiation, λ = 0.71073 Å
Hall symbol: -P 2ybc	Cell parameters from 1017 reflections
*a* = 12.589 (3) Å	θ = 3.0–27.4°
*b* = 8.1026 (17) Å	µ = 6.51 mm^−^^1^
*c* = 25.231 (6) Å	*T* = 293 K
β = 105.174 (2)°	Prism, colourless
*V* = 2483.8 (9) Å^3^	0.61 × 0.15 × 0.12 mm
*Z* = 4	

### Data collection

**Table:**

Bruker SMART APEX CCD area-detector diffractometer	6143 independent reflections
Radiation source: fine-focus sealed tube	5049 reflections with *I* > 2σ(*I*)
Graphite monochromator	*R*_int_ = 0.034
ω scans	θ_max_ = 28.3°, θ_min_ = 2.0°
Absorption correction: multi-scan (*SADABS*; Bruker, 2008)	*h* = −16→16
*T*_min_ = 0.109, *T*_max_ = 0.499	*k* = −10→10
29804 measured reflections	*l* = −33→33

### Refinement

**Table:**

Refinement on *F*^2^	Primary atom site location: structure-invariant direct methods
Least-squares matrix: full	Secondary atom site location: difference Fourier map
*R*[*F*^2^ > 2σ(*F*^2^)] = 0.025	Hydrogen site location: inferred from neighbouring sites
*wR*(*F*^2^) = 0.067	H-atom parameters constrained
*S* = 1.03	*w* = 1/[σ^2^(*F*_o_^2^) + (0.034*P*)^2^ + 0.6725*P*] where *P* = (*F*_o_^2^ + 2*F*_c_^2^)/3
6143 reflections	(Δ/σ)_max_ = 0.002
307 parameters	Δρ_max_ = 0.96 e Å^−^^3^
0 restraints	Δρ_min_ = −0.45 e Å^−^^3^
0 constraints	

### Fractional atomic coordinates and isotropic or equivalent isotropic displacement parameters (Å^2^)

**Table:**

	*x*	*y*	*z*	*U*_iso_*/*U*_eq_	
Hg1	0.394304 (9)	0.004646 (12)	0.767518 (5)	0.04050 (5)	
Cl1	0.48115 (6)	−0.31360 (8)	0.77525 (3)	0.04764 (18)	
Cl2	0.22022 (7)	−0.03302 (11)	0.70223 (4)	0.0586 (2)	
P1	0.33345 (5)	0.13556 (8)	0.87895 (3)	0.02688 (13)	
C1	0.6575 (2)	0.0932 (3)	0.87894 (10)	0.0329 (6)	
C2	0.7437 (2)	0.2055 (4)	0.89500 (11)	0.0382 (6)	
H2A	0.7295	0.3174	0.8977	0.046*	
C3	0.8496 (2)	0.1491 (5)	0.90675 (12)	0.0481 (8)	
C4	0.8745 (3)	−0.0152 (5)	0.90283 (18)	0.0634 (11)	
H4A	0.9473	−0.0502	0.9108	0.076*	
C5	0.7897 (3)	−0.1265 (5)	0.88698 (16)	0.0644 (10)	
H5A	0.8050	−0.2379	0.8841	0.077*	
C6	0.6810 (2)	−0.0729 (4)	0.87524 (13)	0.0474 (7)	
H6A	0.6239	−0.1487	0.8649	0.057*	
C7	0.54244 (19)	0.1632 (3)	0.86703 (10)	0.0289 (5)	
N1	0.9400 (2)	0.2703 (5)	0.92431 (12)	0.0708 (10)	
O1	0.9181 (2)	0.4070 (5)	0.93769 (13)	0.0896 (9)	
O2	1.0322 (2)	0.2238 (5)	0.92538 (17)	0.1280 (14)	
O3	0.53055 (15)	0.3129 (2)	0.86724 (8)	0.0414 (4)	
C8	0.4477 (2)	0.0510 (3)	0.85832 (11)	0.0306 (5)	
H8A	0.4705	−0.0531	0.8777	0.037*	
C9	0.24626 (19)	0.2639 (3)	0.82721 (10)	0.0293 (5)	
C10	0.2909 (2)	0.3735 (3)	0.79627 (11)	0.0387 (6)	
H10A	0.3668	0.3864	0.8039	0.046*	
C11	0.2228 (3)	0.4628 (4)	0.75433 (14)	0.0481 (8)	
H11A	0.2530	0.5347	0.7335	0.058*	
C12	0.1103 (3)	0.4461 (5)	0.74316 (14)	0.0510 (8)	
H12A	0.0648	0.5048	0.7143	0.061*	
C13	0.0650 (2)	0.3422 (4)	0.77476 (13)	0.0502 (8)	
H13A	−0.0111	0.3340	0.7678	0.060*	
C14	0.1319 (2)	0.2502 (4)	0.81666 (12)	0.0406 (6)	
H14A	0.1010	0.1798	0.8376	0.049*	
C15	0.2511 (2)	−0.0368 (3)	0.89107 (11)	0.0301 (5)	
C16	0.2028 (3)	−0.1434 (4)	0.84876 (12)	0.0525 (8)	
H16A	0.2127	−0.1269	0.8139	0.063*	
C17	0.1398 (3)	−0.2744 (4)	0.85828 (14)	0.0594 (9)	
H17A	0.1082	−0.3461	0.8297	0.071*	
C18	0.1233 (2)	−0.2999 (4)	0.90891 (13)	0.0476 (7)	
H18A	0.0806	−0.3880	0.9150	0.057*	
C19	0.1707 (3)	−0.1941 (4)	0.95080 (13)	0.0527 (8)	
H19A	0.1596	−0.2107	0.9854	0.063*	
C20	0.2344 (2)	−0.0633 (4)	0.94231 (12)	0.0428 (6)	
H20A	0.2661	0.0072	0.9712	0.051*	
C21	0.37502 (19)	0.2448 (3)	0.94318 (10)	0.0307 (5)	
C22	0.2989 (2)	0.3465 (3)	0.95842 (12)	0.0449 (7)	
H22A	0.2315	0.3684	0.9337	0.054*	
C23	0.3237 (3)	0.4147 (4)	1.01033 (13)	0.0549 (8)	
H23A	0.2719	0.4803	1.0206	0.066*	
C24	0.4236 (3)	0.3871 (4)	1.04692 (12)	0.0501 (8)	
H24A	0.4398	0.4347	1.0817	0.060*	
C25	0.4997 (3)	0.2890 (4)	1.03198 (11)	0.0438 (7)	
H25A	0.5680	0.2715	1.0566	0.053*	
C26	0.4759 (2)	0.2155 (3)	0.98068 (12)	0.0358 (6)	
H26A	0.5273	0.1467	0.9713	0.043*	

### Atomic displacement parameters (Å^2^)

**Table:**

	*U*^11^	*U*^22^	*U*^33^	*U*^12^	*U*^13^	*U*^23^
Hg1	0.04069 (8)	0.04342 (8)	0.03615 (8)	−0.00409 (4)	0.00785 (5)	−0.00748 (4)
Cl1	0.0525 (4)	0.0346 (3)	0.0604 (5)	0.0059 (3)	0.0229 (4)	−0.0059 (3)
Cl2	0.0417 (4)	0.0791 (6)	0.0487 (5)	−0.0070 (4)	0.0004 (4)	−0.0165 (4)
P1	0.0237 (3)	0.0288 (3)	0.0275 (3)	−0.0013 (2)	0.0054 (2)	−0.0014 (2)
C1	0.0253 (12)	0.0462 (15)	0.0272 (13)	0.0017 (11)	0.0069 (10)	0.0012 (11)
C2	0.0293 (13)	0.0553 (17)	0.0315 (14)	−0.0073 (12)	0.0108 (11)	−0.0036 (12)
C3	0.0293 (15)	0.082 (2)	0.0348 (16)	−0.0070 (15)	0.0119 (12)	−0.0037 (15)
C4	0.0338 (18)	0.095 (3)	0.061 (2)	0.0151 (17)	0.0117 (17)	−0.0031 (18)
C5	0.046 (2)	0.068 (2)	0.077 (3)	0.0197 (18)	0.0127 (18)	−0.0060 (19)
C6	0.0343 (16)	0.0497 (18)	0.056 (2)	0.0063 (13)	0.0084 (14)	−0.0019 (15)
C7	0.0246 (12)	0.0341 (13)	0.0283 (12)	0.0001 (10)	0.0076 (10)	−0.0031 (10)
N1	0.0300 (15)	0.130 (3)	0.0531 (18)	−0.0224 (18)	0.0115 (13)	−0.0100 (19)
O1	0.0589 (18)	0.114 (3)	0.094 (2)	−0.0417 (19)	0.0161 (15)	−0.026 (2)
O2	0.0335 (15)	0.190 (4)	0.165 (3)	−0.0242 (19)	0.0340 (18)	−0.030 (3)
O3	0.0337 (10)	0.0352 (10)	0.0523 (12)	−0.0018 (8)	0.0060 (9)	−0.0041 (9)
C8	0.0257 (12)	0.0305 (11)	0.0341 (14)	0.0010 (10)	0.0053 (10)	−0.0003 (11)
C9	0.0278 (12)	0.0310 (12)	0.0277 (12)	0.0008 (10)	0.0047 (10)	−0.0025 (10)
C10	0.0317 (14)	0.0430 (15)	0.0408 (15)	0.0027 (11)	0.0086 (11)	0.0073 (12)
C11	0.053 (2)	0.0488 (17)	0.0434 (18)	0.0089 (14)	0.0141 (15)	0.0111 (14)
C12	0.0468 (19)	0.0555 (17)	0.0416 (17)	0.0169 (15)	−0.0043 (14)	0.0077 (15)
C13	0.0282 (14)	0.0584 (19)	0.0552 (19)	0.0045 (13)	−0.0045 (13)	−0.0003 (15)
C14	0.0278 (13)	0.0440 (15)	0.0476 (17)	−0.0006 (11)	0.0057 (12)	0.0014 (13)
C15	0.0272 (13)	0.0322 (12)	0.0307 (14)	−0.0001 (10)	0.0074 (11)	0.0013 (10)
C16	0.071 (2)	0.0544 (18)	0.0366 (16)	−0.0258 (16)	0.0225 (15)	−0.0101 (14)
C17	0.073 (2)	0.0522 (19)	0.058 (2)	−0.0321 (17)	0.0262 (18)	−0.0163 (16)
C18	0.0460 (17)	0.0375 (15)	0.065 (2)	−0.0062 (13)	0.0236 (15)	0.0048 (14)
C19	0.063 (2)	0.0566 (19)	0.0454 (18)	−0.0115 (16)	0.0258 (16)	0.0068 (15)
C20	0.0475 (17)	0.0475 (16)	0.0347 (15)	−0.0081 (14)	0.0130 (13)	−0.0026 (13)
C21	0.0286 (12)	0.0320 (12)	0.0313 (13)	−0.0033 (10)	0.0075 (10)	−0.0024 (10)
C22	0.0412 (16)	0.0441 (16)	0.0461 (17)	0.0063 (13)	0.0058 (13)	−0.0101 (13)
C23	0.070 (2)	0.0463 (18)	0.051 (2)	0.0108 (16)	0.0193 (17)	−0.0132 (15)
C24	0.077 (2)	0.0396 (16)	0.0308 (15)	−0.0081 (15)	0.0081 (15)	−0.0078 (12)
C25	0.0507 (17)	0.0447 (16)	0.0293 (15)	−0.0093 (13)	−0.0016 (13)	0.0037 (12)
C26	0.0350 (14)	0.0375 (14)	0.0326 (13)	−0.0003 (11)	0.0045 (11)	0.0016 (12)

### Geometric parameters (Å, º)

**Table:**

Hg1—C8	2.244 (3)	C11—C12	1.375 (5)
Hg1—Cl2	2.3916 (9)	C11—H11A	0.9300
Hg1—Cl1^i^	2.5840 (8)	C12—C13	1.381 (5)
Hg1—Cl1	2.7876 (8)	C12—H12A	0.9300
P1—C8	1.789 (3)	C13—C14	1.384 (4)
P1—C21	1.800 (3)	C13—H13A	0.9300
P1—C9	1.800 (2)	C14—H14A	0.9300
P1—C15	1.812 (3)	C15—C20	1.380 (4)
C1—C6	1.386 (5)	C15—C16	1.384 (4)
C1—C2	1.393 (4)	C16—C17	1.383 (4)
C1—C7	1.511 (3)	C16—H16A	0.9300
C2—C3	1.366 (4)	C17—C18	1.362 (4)
C2—H2A	0.9300	C17—H17A	0.9300
C3—C4	1.377 (5)	C18—C19	1.370 (4)
C3—N1	1.480 (4)	C18—H18A	0.9300
C4—C5	1.374 (5)	C19—C20	1.379 (4)
C4—H4A	0.9300	C19—H19A	0.9300
C5—C6	1.392 (4)	C20—H20A	0.9300
C5—H5A	0.9300	C21—C26	1.391 (4)
C6—H6A	0.9300	C21—C22	1.392 (4)
C7—O3	1.222 (3)	C22—C23	1.380 (4)
C7—C8	1.470 (3)	C22—H22A	0.9300
N1—O1	1.211 (4)	C23—C24	1.370 (5)
N1—O2	1.214 (4)	C23—H23A	0.9300
C8—H8A	0.9800	C24—C25	1.371 (4)
C9—C10	1.394 (4)	C24—H24A	0.9300
C9—C14	1.398 (3)	C25—C26	1.384 (4)
C10—C11	1.380 (4)	C25—H25A	0.9300
C10—H10A	0.9300	C26—H26A	0.9300
			
C8—Hg1—Cl2	134.44 (7)	C12—C11—C10	120.3 (3)
C8—Hg1—Cl1^i^	106.18 (7)	C12—C11—H11A	119.8
Cl2—Hg1—Cl1^i^	109.38 (3)	C10—C11—H11A	119.8
C8—Hg1—Cl1	94.37 (7)	C11—C12—C13	120.0 (3)
Cl2—Hg1—Cl1	101.66 (3)	C11—C12—H12A	120.0
Cl1^i^—Hg1—Cl1	106.661 (16)	C13—C12—H12A	120.0
Hg1^ii^—Cl1—Hg1	140.15 (3)	C12—C13—C14	120.6 (3)
C8—P1—C21	112.58 (12)	C12—C13—H13A	119.7
C8—P1—C9	113.21 (12)	C14—C13—H13A	119.7
C21—P1—C9	110.32 (12)	C13—C14—C9	119.5 (3)
C8—P1—C15	107.04 (12)	C13—C14—H14A	120.2
C21—P1—C15	105.74 (12)	C9—C14—H14A	120.2
C9—P1—C15	107.48 (12)	C20—C15—C16	118.8 (3)
C6—C1—C2	119.3 (2)	C20—C15—P1	120.8 (2)
C6—C1—C7	124.2 (2)	C16—C15—P1	120.4 (2)
C2—C1—C7	116.5 (2)	C17—C16—C15	120.1 (3)
C3—C2—C1	119.1 (3)	C17—C16—H16A	120.0
C3—C2—H2A	120.5	C15—C16—H16A	120.0
C1—C2—H2A	120.5	C18—C17—C16	120.9 (3)
C2—C3—C4	122.4 (3)	C18—C17—H17A	119.5
C2—C3—N1	118.2 (3)	C16—C17—H17A	119.5
C4—C3—N1	119.4 (3)	C17—C18—C19	119.1 (3)
C5—C4—C3	118.8 (3)	C17—C18—H18A	120.4
C5—C4—H4A	120.6	C19—C18—H18A	120.4
C3—C4—H4A	120.6	C18—C19—C20	120.9 (3)
C4—C5—C6	120.1 (3)	C18—C19—H19A	119.5
C4—C5—H5A	119.9	C20—C19—H19A	119.5
C6—C5—H5A	119.9	C19—C20—C15	120.2 (3)
C1—C6—C5	120.3 (3)	C19—C20—H20A	119.9
C1—C6—H6A	119.9	C15—C20—H20A	119.9
C5—C6—H6A	119.9	C26—C21—C22	119.0 (2)
O3—C7—C8	121.3 (2)	C26—C21—P1	121.8 (2)
O3—C7—C1	118.9 (2)	C22—C21—P1	118.75 (19)
C8—C7—C1	119.7 (2)	C23—C22—C21	119.9 (3)
O1—N1—O2	124.3 (4)	C23—C22—H22A	120.1
O1—N1—C3	118.5 (3)	C21—C22—H22A	120.1
O2—N1—C3	117.2 (4)	C24—C23—C22	121.0 (3)
C7—C8—P1	113.71 (18)	C24—C23—H23A	119.5
C7—C8—Hg1	105.71 (17)	C22—C23—H23A	119.5
P1—C8—Hg1	108.21 (12)	C23—C24—C25	119.6 (3)
C7—C8—H8A	109.7	C23—C24—H24A	120.2
P1—C8—H8A	109.7	C25—C24—H24A	120.2
Hg1—C8—H8A	109.7	C24—C25—C26	120.7 (3)
C10—C9—C14	119.3 (2)	C24—C25—H25A	119.6
C10—C9—P1	121.04 (19)	C26—C25—H25A	119.6
C14—C9—P1	119.6 (2)	C25—C26—C21	119.8 (3)
C11—C10—C9	120.2 (3)	C25—C26—H26A	120.1
C11—C10—H10A	119.9	C21—C26—H26A	120.1
C9—C10—H10A	119.9		
			
C8—Hg1—Cl1—Hg1^ii^	−130.02 (8)	C8—P1—C9—C14	137.1 (2)
Cl2—Hg1—Cl1—Hg1^ii^	92.86 (5)	C21—P1—C9—C14	−95.7 (2)
Cl1^i^—Hg1—Cl1—Hg1^ii^	−21.69 (4)	C15—P1—C9—C14	19.1 (2)
C6—C1—C2—C3	0.0 (4)	C14—C9—C10—C11	−2.4 (4)
C7—C1—C2—C3	179.5 (2)	P1—C9—C10—C11	175.7 (2)
C1—C2—C3—C4	0.6 (4)	C9—C10—C11—C12	0.8 (5)
C1—C2—C3—N1	−179.4 (3)	C10—C11—C12—C13	1.4 (5)
C2—C3—C4—C5	−0.6 (6)	C11—C12—C13—C14	−2.1 (5)
N1—C3—C4—C5	179.4 (3)	C12—C13—C14—C9	0.5 (5)
C3—C4—C5—C6	−0.1 (6)	C10—C9—C14—C13	1.7 (4)
C2—C1—C6—C5	−0.6 (4)	P1—C9—C14—C13	−176.4 (2)
C7—C1—C6—C5	180.0 (3)	C8—P1—C15—C20	118.7 (2)
C4—C5—C6—C1	0.6 (5)	C21—P1—C15—C20	−1.6 (3)
C6—C1—C7—O3	−174.2 (2)	C9—P1—C15—C20	−119.4 (2)
C2—C1—C7—O3	6.3 (4)	C8—P1—C15—C16	−62.0 (3)
C6—C1—C7—C8	9.0 (4)	C21—P1—C15—C16	177.8 (2)
C2—C1—C7—C8	−170.5 (2)	C9—P1—C15—C16	59.9 (3)
C2—C3—N1—O1	13.9 (5)	C20—C15—C16—C17	−0.5 (5)
C4—C3—N1—O1	−166.0 (4)	P1—C15—C16—C17	−179.8 (3)
C2—C3—N1—O2	−167.6 (3)	C15—C16—C17—C18	0.6 (5)
C4—C3—N1—O2	12.4 (5)	C16—C17—C18—C19	−0.3 (5)
O3—C7—C8—P1	−26.8 (3)	C17—C18—C19—C20	−0.1 (5)
C1—C7—C8—P1	149.97 (19)	C18—C19—C20—C15	0.2 (5)
O3—C7—C8—Hg1	91.8 (2)	C16—C15—C20—C19	0.1 (4)
C1—C7—C8—Hg1	−91.5 (2)	P1—C15—C20—C19	179.4 (2)
C21—P1—C8—C7	−42.2 (2)	C8—P1—C21—C26	−21.5 (3)
C9—P1—C8—C7	83.8 (2)	C9—P1—C21—C26	−149.0 (2)
C15—P1—C8—C7	−157.94 (19)	C15—P1—C21—C26	95.0 (2)
C21—P1—C8—Hg1	−159.28 (11)	C8—P1—C21—C22	166.2 (2)
C9—P1—C8—Hg1	−33.30 (16)	C9—P1—C21—C22	38.7 (2)
C15—P1—C8—Hg1	84.95 (14)	C15—P1—C21—C22	−77.2 (2)
Cl2—Hg1—C8—C7	−143.68 (13)	C26—C21—C22—C23	−0.8 (4)
Cl1^i^—Hg1—C8—C7	−3.47 (17)	P1—C21—C22—C23	171.7 (2)
Cl1—Hg1—C8—C7	105.29 (15)	C21—C22—C23—C24	1.6 (5)
Cl2—Hg1—C8—P1	−21.53 (18)	C22—C23—C24—C25	−0.7 (5)
Cl1^i^—Hg1—C8—P1	118.69 (11)	C23—C24—C25—C26	−1.0 (5)
Cl1—Hg1—C8—P1	−132.55 (11)	C24—C25—C26—C21	1.7 (4)
C8—P1—C9—C10	−40.9 (3)	C22—C21—C26—C25	−0.8 (4)
C21—P1—C9—C10	86.2 (2)	P1—C21—C26—C25	−173.1 (2)
C15—P1—C9—C10	−158.9 (2)		

Symmetry codes: (i) −*x*+1, *y*+1/2, −*z*+3/2; (ii) −*x*+1, *y*−1/2, −*z*+3/2.

### Hydrogen-bond geometry (Å, º)

**Table:**

*D*—H···*A*	*D*—H	H···*A*	*D*···*A*	*D*—H···*A*
C10—H10*A*···O3	0.93	2.33	3.118 (3)	142
C6—H6*A*···Cl1	0.93	2.83	3.630 (3)	145
C24—H24*A*···O3^iii^	0.93	2.39	3.206 (3)	146

Symmetry code: (iii) −*x*+1, −*y*+1, −*z*+2.
